# MiR-409-5p as a Regulator of Neurite Growth Is Down Regulated in APP/PS1 Murine Model of Alzheimer’s Disease

**DOI:** 10.3389/fnins.2019.01264

**Published:** 2019-11-28

**Authors:** Jing Guo, Yifei Cai, Xiaoyang Ye, Nana Ma, Yuan Wang, Bo Yu, Jun Wan

**Affiliations:** ^1^Shenzhen Key Laboratory for Neuronal Structural Biology, Biomedical Research Institute, Shenzhen Peking University – The Hong Kong University of Science and Technology Medical Center, Shenzhen, China; ^2^Shenzhen Key Laboratory for Translational Medicine of Dermatology, Biomedical Research Institute, Shenzhen Peking University – The Hong Kong University of Science and Technology Medical Center, Shenzhen, China; ^3^Department of Dermatology, Peking University Shenzhen Hospital, Shenzhen, China; ^4^Division of Life Science, The Hong Kong University of Science and Technology, Hong Kong, China

**Keywords:** Alzheimer’s disease, beta amyloid peptide, microRNA, mir-409-5p, Plek

## Abstract

Alzheimer’s disease (AD) is a heterogeneous neurodegenerative disease. Recent studies suggest that miRNA expression changes are associated with the development of AD. Our previous study showed that the expression level of miR-409-5p was stably downregulated in the early stage of APP/PS1 double transgenic mice model of AD. We now report that miR-409-5p impairs neurite outgrowth, decreases neuronal viability, and accelerates the progression of Aβ_1__–__42_-induced pathologies. In this study, we found that Aβ_1__–__42_ peptide significantly decreased the expression of miR-409-5p, which was consistent with the expression profile of miR-409-5p in the APP/PS1 mice cortexes. Plek was confirmed to be a potential regulatory target of miR-409-5p by luciferase assay and Western blotting. Overexpression of miR-409-5p has an obvious neurotoxicity in neuronal cell viability and differentiation, whereas Plek overexpression could partially rescue neurite outgrowth from this toxicity. Some cytoskeleton regulatory proteins have been found to be related to AD pathogenesis. Our data show some clues that cytoskeletal reorganization may play roles in AD pathology. The early downregulation of miR-409-5p in AD progression might be a self-protective reaction to alleviate the synaptic damage induced by Aβ, which may be used as a potential early biomarker of AD.

## Introduction

Alzheimer’s disease (AD) is the most common cause of dementia in the elderly. It is an irreversible neurodegenerative disorder, with the hallmarks of senile plaques, neurofibrillary tangles (NFTs), and neuronal loss (Barker et al., 2002; Vinters, 2015). Clinical symptoms of AD include loss of recent memory, faulty judgment, personality changes, and progressive loss of reasoning power (Robakis, 2011). Although it has been widely explored, the exact pathogenesis of AD remains to be elucidated.

MicroRNAs (miRs) are double-stranded RNAs that play regulatory roles in protein expression (Iwakawa and Tomari, 2015). miRs-regulated protein expression plays important roles in AD pathogenesis, and miRs have the potential to be a more sensitive approach for detection and management of AD (Basavaraju and de Lencastre, 2016; Gupta et al., 2017; Reddy et al., 2017). Cytoskeletal abnormalities and synaptic impairment are typical in amyloid β (Aβ)-induced stress (Bamburg and Bernstein, 2016). miRs have the ability to regulate cytoskeletal changes in many diseases especially cancers (Gross, 2013; Kanakkanthara and Miller, 2013; Zhang et al., 2019).

Cytoskeleton plays a vital role in the maintenance of the nervous system through adulthood. The changes of neurofilaments and microtubule-associated proteins have been linked to a variety of neurodegenerative diseases (Bettencourt da Cruz et al., 2005). At early stages of AD, it has been known that cytoskeleton is disrupted in neurons. Tau protein is hyperphosphorylated in AD, which results in tangle formation and abnormal microtubule assembly (Lindwall and Cole, 1984; Alonso et al., 1996). Besides tau, many other proteins involved in the regulation of cytoskeleton might be related to AD pathogenesis (Bamburg and Bernstein, 2016; Brandt and Bakota, 2017). Our previous studies also showed that miRNAs that regulated neurite outgrowth were also involved in Aβ-induced neuronal impairment (Ye et al., 2015; Fan et al., 2016). The whole transcriptome sequencing showed that some cytoskeleton-related proteins were upregulated in cortexes of APPswe/PS1ΔE9 double transgenic mice (APP/PS1 mice) when compared with age-matched control mice (NCBI GEO database: GSE87550), including RAS-like family 12 (Rasl12), pleckstrin (Plek), and syndecan binding protein 2 (Sdcbp2). Plek could induce cytoskeletal reorganization *via* a rac-dependent pathway (Ma and Abrams, 1999; Baig et al., 2009). Syndecan family members, in concert with Sdcbp2 (Syndecan Binding Protein 2, Syntenin2), may mediate apoE-dependent neurite outgrowth by interacting with actin filament (Zimmermann et al., 2005; Kim et al., 2014).

In this study, we investigate the role of miR-409-5p in neurite outgrowth regulation by targeting Plek, which may contribute to the synaptic failure and cognitive dysfunction in AD.

## Materials and Methods

### Reagents

Rabbit polyclonal antibodies against Plek (12506-1-AP) and SDCBP2 (10407-1-AP) were from ProteinTech Company. Rabbit polyclonal antibody against α-tubulin (#2144) was from Cell Signaling Technologies. Mouse monoclonal antibody against β-tubulin III (T8575), the secondary goat anti-rabbit IgG antibody (A9169), and Aβ_1__–_42 (03111) were from Sigma. The mimic or inhibitors of miR-409-5p were synthesized by RiboBio Company.

### Plasmid Construction

The 3′UTR fragments of mouse Plek and Sdcbp2 were amplified by PCR from a mouse cDNA library. PCR amplicon was cloned into psiCHECK2 vector between *Xho*I and *Not*I sites using a ClonExpress^®^ II One Step Cloning Kit (Vazyme). For luciferase activity assay, we introduced mutations on each miR-409-5p miR binding site by overlap PCR. Mouse Plek cDNA was amplified by PCR and cloned into PTGFP vector (a modified vector form pEGFP-C1) by *Xho*I and *Eco*RI sites. All of the primer sequences were shown in Table 1. The sequences of all constructs were confirmed by DNA sequencing.

**TABLE 1:** Sequences of synthesized miR mimics/inhibitors or siRNA.

**Primer name**	**Primer sequence**
WT-Plek1-F	5′-aattctaggcgatcgctcgagGGAAGAAGCATAAGGATGGA CATC-3′
WT-Plek1-R	5′-attttattgcggccagcggccgcCACACAGTGTTCAGCACT GGTCT-3′
WT-Plek2-F	5′-aattctaggcgatcgctcgagACTGAAAACAACTTCTTCTAAGTTAT TTCTG-3′
WT-Plek2-R	5′-attttattgcggccagcggccgcAACAATAGAAATACCATTCCTGA CAGG-3′
WT-Sdcbp2-F	5′-aattctaggcgatcgctcgagTATTGCAGGGATGTGTCCCAG-3′
WT-Sdcbp2-R	5′-attttattgcggccagcggccgcATCCACTCTTTATTCAAAAGAC AGTGA-3′
Mut-plek1-F	5′-TATACGCAGATTGGTATATCACAGTGG-3′
Mut-plek1-R	5′-AATCTGCGTATATGGGTAACCATTC-3′
Mut-plek2-F	5′-GGGTACGCTGAATGGCTTTCTCATAG-3′
Mut-plek2-R	5′-TTCAGCGTACCCCTACAAGTAATG-3′
Mut-sdcbp2-F	5′-ATAGCGCTACTTCACTGTCTTTTG-3′
Mut-sdcbp2-R	5′-AAGTAGCGCTATTAGTCTGTACATAG-3′
Plek-GFP-F	5′-gatctcgacggatccgaattcGAACCAAAGCGGATCAGGGC-3′
Plek-GFP-R	5′-agatccggtggatcgctcgagTCATTTCCCAGTCCGTGAGG-3′

### Animals

The APPswe/PS1ΔE9 double transgenic mice contained two mutated human genes, APP695 with Swedish mutation K595N/M596L and Presenilin1 without exon9 (PS1ΔE9). The mice originally came from the Jackson Laboratory (Borchelt et al., 1996) and purchased from the Model Animal Research Center of Nanjing University (Nanjing, China). The experimental protocol, approved by the Committee for the Ethics of Animal Experiments, Shenzhen-Peking University-The Hong Kong University of Science and Technology Medical Center (SPHMC) (protocol number 2011-004), was performed as described before (Wan et al., 2010).

### Cell Cultures and Transfection

PC12 cells, Neuro2A cells, and HEK293T cells were cultured as described before (Fan et al., 2016; Wu et al., 2016). Primary hippocampal neurons were prepared from embryonic day 18.5 embryos of C57B6 mice. The hippocampi were dissected, minced, and trypsinized. Neurons were seeded on Petri dishes coated by poly-D-lysine (Sigma) and cultured in neurobasal medium (Life Technologies) containing 2% B27 (Life Technologies) and 0.5 mM L-glutamine in a humidified incubator at 37°C with 5% CO_2_. PC12 cells, Neuro2A cells, or primary hippocampal neurons were transfected with miR-409-5p mimic or inhibitor by ViaFect Transfection Reagent (Promega) according to the manufacturer’s instructions.

### Aβ_1__–__42_ Treatment

Aβ_1__–__42_ (03111, Sigma) was dissolved with 1% ammonium hydroxide by pipette mixing, followed by diluting to 200 μM in PBS. The diluted Aβ_1__–__42_ was incubated at 37°C overnight to induce aggregation before use. The aggregated Aβ_1__–__42_ was diluted to 4–10 μM as the final concentration with culture medium and treated to the cells. Cell toxicity induced by Aβ_1__–__42_ was detected in primary-cultured hippocampal neurons (Supplementary Figure S1).

### Cell Viability Assay

Five thousand hippocampal neurons were plated in each well in a 96-well plate. Two hundred nanomolar of miR-409-5p was transfected. Forty-eight hours after transfection, hippocampal neurons were incubated with CellTiter 96 AQueous (Promega) for 4 h to form insoluble purple formazan. Then, the absorbance at OD_490_ was measured with an ELISA plate reader (BioRad). All experiments were repeated at least three times.

### Luciferase Reporter Assay

293T cells were cotransfected with wild-type (WT) or mutated *psiCHECK-Plek-3*′*UTR* or *psiCHECK-Sdcbp2-3*′*UTR* and miR-409-5p for 24 h. Cell lysate was harvested, and the luciferase reporter gene assay kit (Promega) was used to measure the luciferase activities. All experiments were repeated at least three times.

### RNA Extraction and Real-Time PCR

Total RNA was extracted using TRIzol Reagent (Life Technologies) according to the manufacturer’s instructions. RNA quantity was measured by NanoDrop 2000 (Thermo Fisher Scientific). cDNA was synthesized, and quantitative PCR (qPCR) was performed as described before (Wu et al., 2016). cDNA was synthesized from 100 ng of total RNA by miR-specific RT primers using a Reverse Transcription System (Promega). qPCR was subsequently performed in triplicate with a 1:4 dilution of cDNA using the 2 × SYBR green SuperMix (Bio-Rad) on a CFX96 Touch Real-Time PCR Detection System (Bio-Rad). The expression level of miR-409-5p was normalized against that of U6. Glyceraldehyde-phosphate dehydrogenase (GAPDH) was used as the control of Plek and Sdcbp2 mRNA quantification. Data were collected and analyzed with the Bio-Rad software using 2^–ΔΔCt^ method for quantification of the relative expression levels. The primer sequences were shown in Table 2. All experiments were repeated at least three times.

**TABLE 2:** Sequence of the primers used for real-time PCR.

**Primer name**	**Sequence**
miR-409-5p- RT	5′-GTCGTATCCAGTGCGTGTCGTGGAGTCGGCAA TTGCACTGGATACGACATGCAA-3′
miR-409-5p -F	5′-AGGTTACCCGAGCAACTTTG-3′
miR-409-5p -R	5′-GTGTCGTGGAGTCGGCAA-3′
U6-F	5′-GCTTCGGCAGCACATATACTAAAAAT-3′
U6-R	5′-CGCTTCACGAATTTGCGTGTCAT-3′
Ms-GAPDH-F	5′-AACTTTGGCATTGTGGAAGG-3′
Ms-GAPDH-R	5′-GGATGCAGGGATGATGTTCT-3′
Ms-Sdcbp2-F	5′-AAGAAGGCATCAGCGGAGAA-3′
Ms-Sdcbp2-R	5′-TGTGGTGAAGCAGCAACGGG-3′
Ms-Plek-F	5′-AAAGAAAAGTGACAATAGCCCCA-3′
Ms-Plek-R	5′-CATAGACAGATACAAAGCCCCCA-3′

### Western Blotting

Cells were lysed in RIPA buffer with protease inhibitor cocktail (Thermo Scientific) on ice for half an hour. Brain lysate was harvested and subjected to SDS-PAGE as described before (Wu et al., 2016). Gel-separated proteins were then transferred to NC membrane followed by Western blotting with a primary antibody 1:200 at 4°C overnight and a horseradish peroxidase-conjugated secondary antibody 1:5,000 for 2 h at room temperature. Signals were developed with the Super Signal West Pico Chemiluminescent Substrate (Thermo Scientific). Optical density was quantified by Quantity One (Bio-Rad). ImageJ software was used to quantify the gray degree values.

### Fluorescence Immunostaining

Cells were fixed for 30 min at room temperature in a 4% PFA solution and then permeabilized and blocked in PBS containing 1% BSA, 4% goat serum, and 0.4% Triton X-100 for 30 min at room temperature. The primary antibody (anti-β-tubulin III, 1:500, Sigma, mouse monoclonal antibody) at 4°C was used to incubate cells overnight, followed by fluorescence-conjugated secondary antibody (anti-mouse 555, 1:500, Jackson Immuno Research) for 2 h at room temperature. Finally, cells were stained with DAPI for 5 min, then washed with PBS followed by deionized water and mounted with antifade mounting medium (Beyotime). Images of neurons were captured by a Zeiss LSM 710 confocal microscope with a 40 × objective. Images were analyzed with Zen 2012 (Zeiss) and ImageJ (NIH).

### Quantification of Neurite Outgrowth

In the morphological study of cells, length of the longest neurite and total neurite length were measured to quantify neurite outgrowth using ImageJ software. For each measurement, at least 100 cells were counted from randomly selected fields. Each experiment was repeated at least three times. The total number of tip ends was counted to represent the number of neurites from individual cells.

### Computational Analysis of miR-409-5p Targets

The target genes for miR-409-5p were predicted by four databases: miRDB^1^, mi-Randa^2^, DIANA microT-CDS (v5)^3^, and targetScan^4^. To narrow down the pool of the potential targets, the *p*-value threshold and the microthreshold were set to be 0.05 and 0.7, respectively. Venn tool^5^ was used to take the intersection of the four database target genes. The biological functions and the annotation of KEGG pathways of the intersection genes were analyzed by Gene Ontology^6^ and DIANA, respectively.

### Statistical Analyses

Data are expressed as the mean ±SD. Statistical comparisons were made using SPSS 20.0 software. Unpaired *t*-test was used to analyze differences between two groups, ANOVA followed by *post hoc* analysis using Dunnett’s test was used among multiple groups, and two-way ANOVA was used to analyze differences among different ages of WT and APP/PS1 mice. *P*-value < 0.05 is considered statistically significant.

## Results

### The Expression of miR-409-5p Was Significantly Decreased in Both APP/PS1 Mice and Oligomeric Aβ_1__–__42_-Treated Cells

**FIGURE 1:**
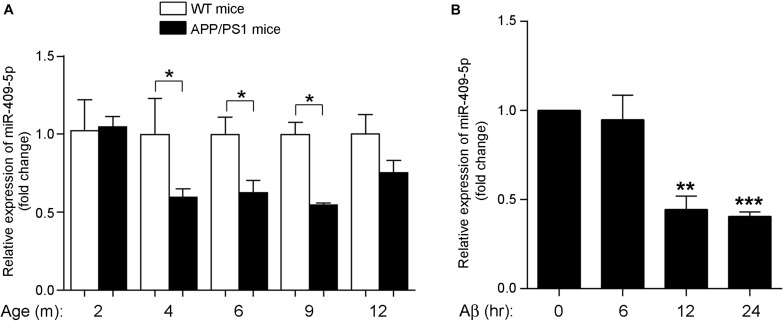
MiR-409-5p was significantly decreased in APP/PS1 mouse and oligomeric Aβ_1–__42_ peptide-treated cells. **(A)** Relative expression levels of miR-409-5p were quantified by RT-qPCR in cortex tissues of APP/PS1 and wild-type (WT) mice from 2 to 12 months (4–6 mice for each age). The levels were normalized to the expression of snRNAU6. The results were shown as the mean ± SD (^∗^*p* < 0.05, *n* = 3). Two-way ANOVA was used to analyze differences. **(B)** PC12 cells had differentiated for 48 h. The diluted Aβ_1–__42_ was incubated at 37°C overnight to induce aggregation, then 4 μM oligomeric Aβ_1–__42_ per well in differentiation medium of PC12 cells. Relative expression level of miR-409-5p was examined by RT-qPCR at different time points. The results were shown as the mean ± SD (^∗∗^*p* < 0.01, ^∗∗∗^*p* < 0.001). The experiment was repeated independently for three times. ANOVA followed by *post hoc* analysis using Dunnett’s test was used to analyze differences.

In the AD mouse model, APP/PS1 double transgenic mice cortexes, we further verified the previous result that the expression level of miR-409-5p was stably downregulated in 4- to 12-month-old APP/PS1 cortexes (Figure 1A). To investigate the role of Aβ_1__–__42_ in miR-409-5p downregulation, we treated Aβ_1__–__42_ to differentiated PC12 cells and found that the expression level of miR-409-5p was reduced significantly after 12-h treatment (Figure 1B), which was consistent with the expression profile in APP/PS1 mice brains.

### MiR-409-5p Reduced Neuronal Survival

**FIGURE 2:**
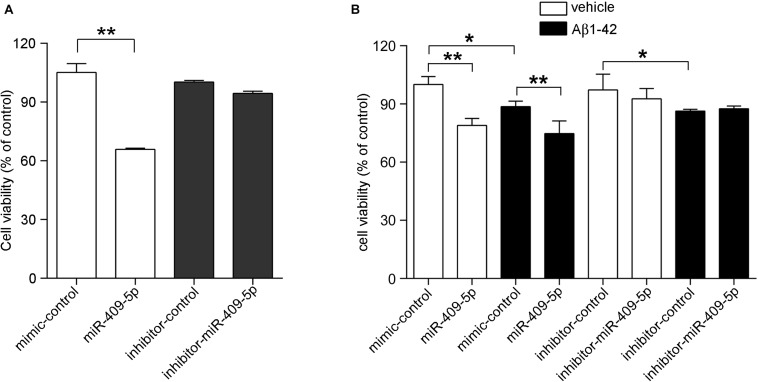
MiR-409-5p and oligomeric Aβ_1–__42_ peptide significantly decreased neuronal viability. **(A)** Mouse primary cultured hippocampal neurons were transfected with miR-409-5p mimic or inhibitor before culturing. Three days later, the MTS/PMS assay was performed to evaluate the cell viability. **(B)** Mouse primary cultured hippocampal neurons were transfected with miR-409-5p mimic or inhibitor. Forty-eight hours later, the cells were treated with 10 μM oligomeric Aβ_1–__42_ for another 24 h. MTS/PMS assay was performed to evaluate the cell viability. The results were shown as the mean ± SD (^∗^*p* < 0.05, ^∗∗^*p* < 0.01). The experiment was repeated independently for three times. Unpaired *t*-test was used to analyze differences.

The overexpression and knocking-down effect of miR-409-5p mimic and inhibitor was shown in Supplementary Figure S2. We transfected miR-409-5p mimic or inhibitor to cultured hippocampal neurons and found that miR-409-5p overexpression significantly reduced the cell viability. However, its inhibitor did not have any effect (Figure 2A). Then, we treated Aβ_1__–__42_ to miR-409-5p-overexpressed or inhibited neurons. It was shown that miR-409-5p mimic aggravated the damage induced by Aβ_1__–__42_, but the miRNA inhibitor cannot rescue from cell death (Figure 2B). This result indicates that miR-409-5p is toxic for neuronal cells, but blocking of this miRNA is not sufficient for cell survival.

### MiR-409-5p Reduced Neurite Outgrowth

**FIGURE 3:**
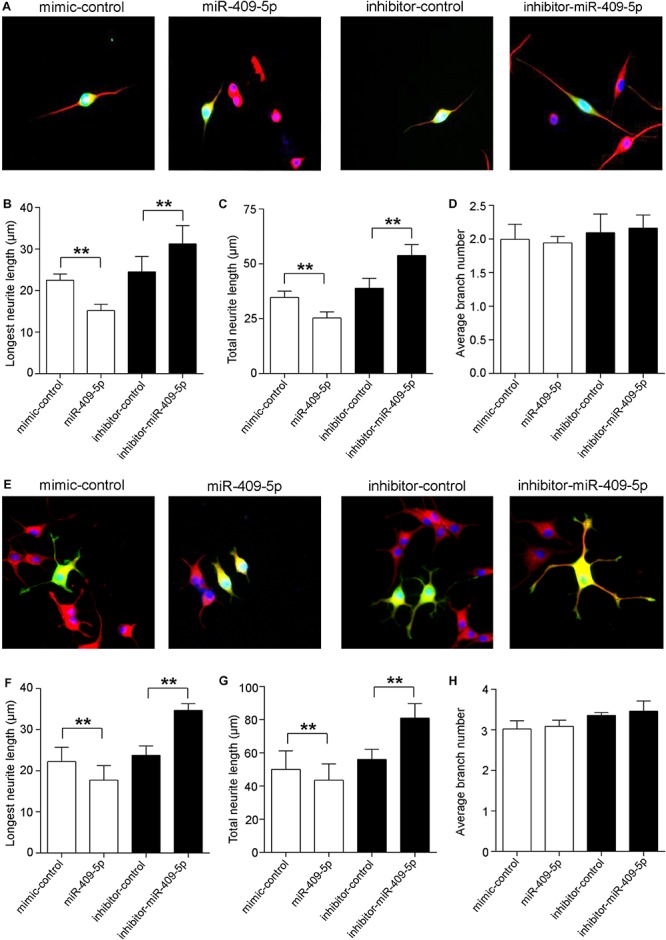
MiR-409-5p slightly aggravated the insult of Aβ_1–__42_ on neurite growth and branching in neuronal-like cell lines. Neuro2A **(A–D)** or PC12 **(E–H)** cells were transfected with PEGFP-C2 (0.5 μg) and miR-409-5p mimic (100 nM) or inhibitor (100 nM). After 3 days, cells were fixed and immunostained with anti-β-tubulin III antibody. The longest neurite length, total neurite length, and branch numbers were quantified of by ImageJ software. The results were shown as the mean ± SD (^∗∗^*p* < 0.01). The experiment was repeated independently for three times. Red, β-tubulin III; Green, GFP; Yellow, merged. Unpaired *t*-test was used to analyze differences.

In order to know the role of miR-409-5p in neuronal differentiation, we tested its expression levels along cell differentiation in two neuron-like cell lines, Neuro2A cells and PC12 cells. Along with RA/NGF-induced cell differentiation, miR-409-5p had a slight increase but without significant difference (Supplementary Figure S3). Then we transfected its mimic or inhibitor to two neuron-like cell lines, Neuro2A cells (Figures 3A–D) and PC12 cells (Figures 3E–H). Overexpression of miR-409-5p significantly decreased the longest neurite length (Figures 3B,F, white bars) and total neurite length (Figures 3C,G, white bars). Consistently, inhibition of miR-409-5p induced the increase of both the longest and total neurite length (Figures 3B,C,F,G, black bars). However, neither mimic nor inhibitor of miR-409-5p can affect the neurite branch numbers (Figures 3D,H).

**FIGURE 4:**
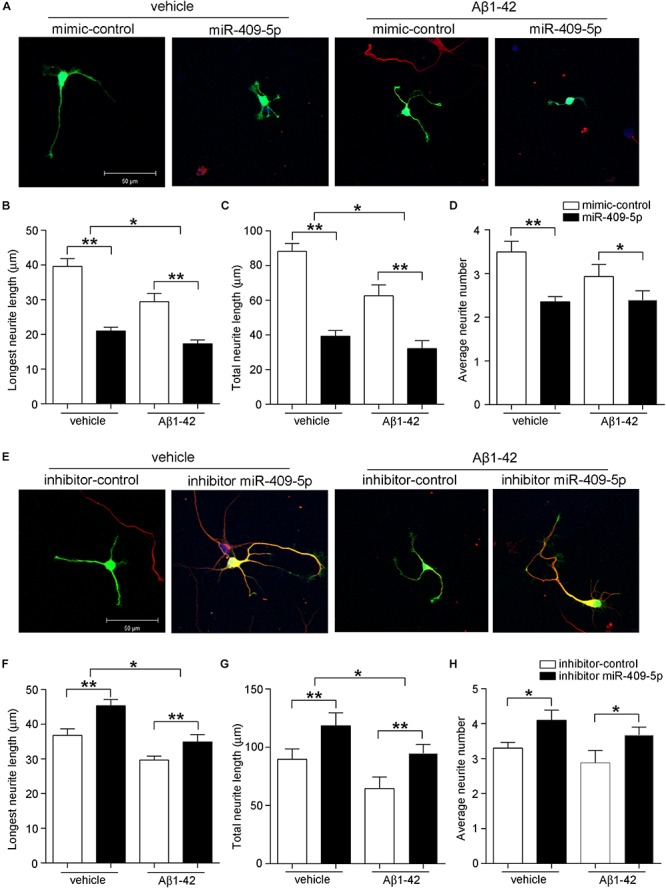
MiR-409-5p aggravated the insult of Aβ_1–__42_ on neurite growth and branching in primary cultured hippocampal neurons. Mouse primary hippocampal neurons were transfected with PEGFP-C2 (0.5 μg) and miR-409-5p mimic (100 nM) **(A–D)** or inhibitor (100 nM) **(E–H)** before culturing, followed by Aβ_1–__42_ treatment (5 μM) or vehicle. After 3 days, cells were fixed and immunostained with anti-β-tubulin III antibody. The longest neurite length, total neurite length, and branch numbers were quantified by ImageJ software. The results were shown as the mean ± SD (^∗^*p* < 0.05,^∗∗^*p* < 0.01). The experiment was repeated independently for three times. Red, β-tubulin III; Green, GFP; Yellow, merged. Unpaired *t*-test was used to analyze differences.

In primary cultured hippocampal neurons, there was a similar effect. MiR-409-5p mimic can decrease both the neurite length and neurite numbers and further aggravated the Aβ_1__–__42_ damage on neurite differentiation (Figures 4A–D). Interestingly, the inhibitor of miR-409-5p can not only increase the neurite outgrowth but also partially rescue the Aβ_1__–__42_-induced neurite impairment (Figures 4E–H), indicating a protective role in Aβ_1__–__42_-related neuronal damage.

### Searching of miR-409-5p Potential Targets

**FIGURE 5:**
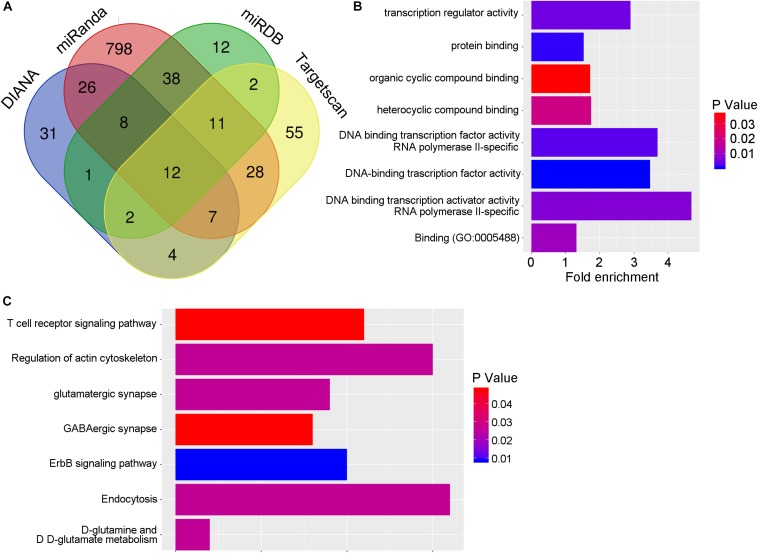
The potential targets of miR-409-5p. **(A)** The target genes of miR-409-5p were hunted by four prediction databases: miRDB, miRanda, DIANA, and TargetScan. There are 139 target genes intersected in more than two databases by Venn tool. **(B,C)** Then the biological functions of the intersection genes were analyzed by Gene Ontology, and the KEGG pathways were predicted by DIANA (Table 3). To further narrow down the pool of the potential targets, the 139 target genes were intersected again with upregulated genes (fold changes >1.2) in APP/PS1 mice by a whole transcriptome sequencing previously done in our laboratory.

To elucidate the mechanisms of miR-409-5p on regulating the neurite outgrowth and neuronal survival, we searched different databases to explore the miR-409-5p target genes (Supplementary Table S1). As shown in Figure 5A, four databases were used in this study, and totally 139 target genes were intersected in more than two databases by Venn tool. The biological functions of the intersection genes were analyzed by Gene Ontology (GO) (Figure 5B), and the KEGG pathways were predicted by DIANA (Figure 5C). The GO analysis showed that target genes mainly participated in posttranscriptional regulation. The KEGG pathway analysis indicated that target genes were enriched in actin cytoskeleton regulation, endocytosis, and ErbB signaling pathway. To further narrow down the pool of the potential targets, among the 139 target genes, those that had more than 1.2-fold upregulated expression in APP/PS1 mice by a whole transcriptome sequencing previously performed (Cai et al., 2017) were selected. Finally, we got nine genes as shown in Table 3, including Rasl12, Gsg2, Tmc5, Plek, Slc26a1, Kcnh8, Sdcbp2, Arap3, and Itgb4. As Plek and sdcbp2 were reported to be involved in the regulation of actin cytoskeleton (Ma and Abrams, 1999; Zimmermann et al., 2005), these two candidates were selected for further study.

**TABLE 3:** Nine genes potential target genes.

**Gene name**	**Description**	**APP/WT Fold changes**
Rasl12	RAS Like Family 12	1.4828
Gsg2	Histone H3 associated protein kinase	2.2721
Tmc5	Transmembrane channel-like gene family 5	2.9189
Plek	Pleckstrin	2.1785
Slc26a1	Solute carrier family 26 (sulfate transporter), member 1	1.2615
Kcnh8	Potassium voltage-gated channel, subfamily, member 8	1.2034
Sdcbp2	Syndecan binding protein (syntenin) 2	1.5324
Arap3	ArfGAP with RhoGAP domain, ankyrin repeat and PH domain 3	1.3185
Itgb4	Integrin beta 4	1.8347

**FIGURE 6:**
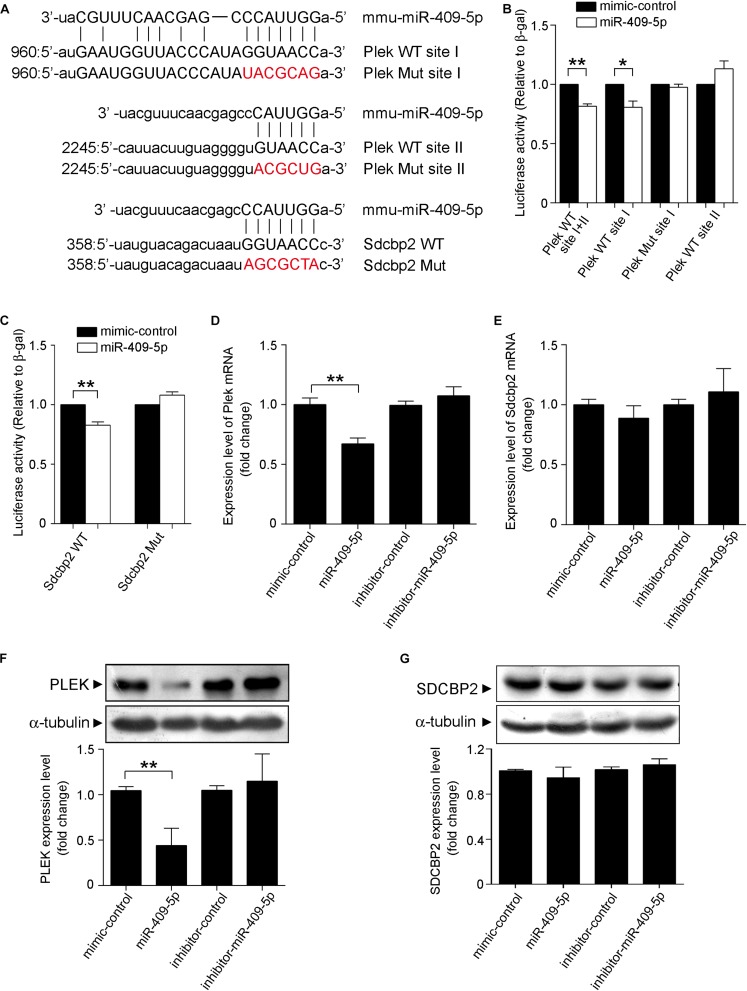
Plek and Sdcbp2 might be the targets of miR-409-5p. **(A)** The wild-type (WT) and mutated plek or Sdcbp2 binding sites with miR-409-5p. **(B)** Relative Renilla/luciferase luminescence of a psiCHECK2 vector construct harboring Plek or mutant Plek cotransfected with miR-409-5p in the HEK 293T cells, with empty psiCHECK2 vector as control. **(C)** Relative Renilla/luciferase luminescence of a psiCHECK2 vector construct harboring Sdcbp2 or mutant Sdcbp2 cotransfected with miR-409-5p in the HEK 293T cells, with empty psiCHECK2 vector as control. The results were shown as the mean ± SD (^∗^*p* < 0.05,^∗∗^*p* < 0.01). The experiment was repeated independently for at least three times. **(D,E)** MiR-409-5p mimic (100 nM) or inhibitor (100 nM) was transfected into Neuro-2a cells. After 24 h, plek and sdcbp2 mRNA levels were assessed by RT-qPCR. **(F,G)** Western blot analysis of Plek and SDCBP2 protein level in primary cortical neurons transfected with miR-409-5p mimic (100 nM) or inhibitor (100 nM) for 72 h. ImageJ software was used to quantify the gray degree values of Plek and SDCBP2. All results were shown as the mean ± SD (^∗^*p* < 0.05). The experiment was repeated independently for three times. Unpaired *t*-test was used to analyze differences.

The 3′UTRs of Plek and Sdcbp2 were analyzed by miRanda database. Database prediction indicated that Plek had two miR-409-5p binding sites in its 3′UTR and Sdcbp2 had one (Figure 6A). To explore the potential role of miR-409-5p in translational regulation of target genes, we cotransfected a luciferase reporter construct containing different fragments of Plek 3′UTR or Sdcbp2 3′UTR together with miR-409-5p. Cotransfection of miR-409-5p resulted in a decrease in luciferase activity when with the expression of Plek 3′UTR full-length fragment containing site I and Sdcbp2 3′UTR (Figures 6B,C). We also generated site-directed mutations on Plek 3′UTR site I and Sdcbp2 3′UTR, and the luciferase activity inhibition disappeared. We found that miR-409-5p significantly suppressed Plek mRNA and protein expression level in primary cultured mouse hippocampal neurons (Figures 6D,F), while it had no effect on Sdcbp2 expression (Figures 6E,G). Our results indicated that Plek might be one of the miR-409-5p target genes.

### Plek Is Upregulated in AD Mice Brains and Rescues miR-409-5p-Induced Neurite Impairment

**FIGURE 7:**
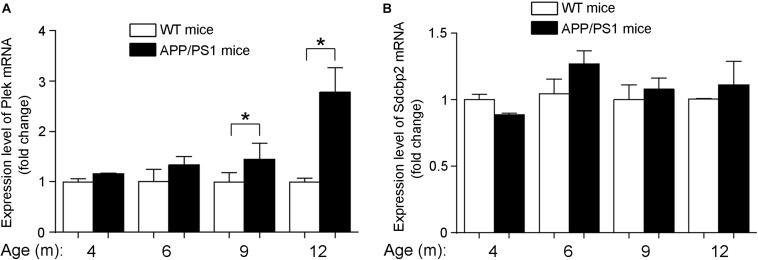
Expression levels of Plek and Sdcbp2 in APP/PS1 cortex. **(A,B)** The relative expression levels of plek and sdcbp2 were examined by RT-qPCR analysis in 4- to 12-month-old APP/PS1 and WT mice cortices (4–6 mice for each age). Two-way ANOVA was used to analyze differences. ^∗^*p* < 0.05.

**FIGURE 8:**
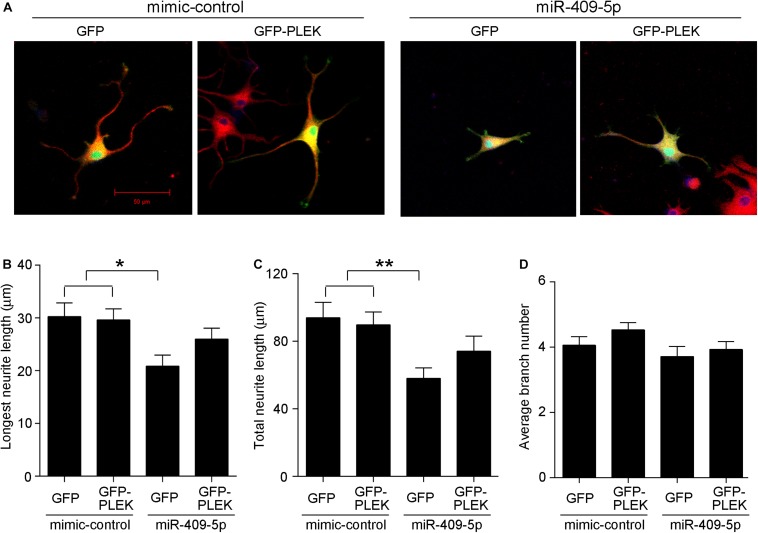
**(A)** Overexpression of Plek partially rescues miR-409-5p-induced neurite outgrowth inhibition. PC12 cells were cotransfected with miR-409-5p mimic or inhibitor (concentration ratio 1:5) with or without PEGFP-Plek before differentiation. After 48 h, cells were fixed and immunostained with anti-β-tubulin III antibody. **(B–D)** Quantification of neurite length or branches was analyzed by ImageJ software. The results were shown as the mean ± SD (^∗^*p* < 0.05, ^∗∗^*p* < 0.01). The experiment was repeated independently for three times. Red, β-tubulin III; Green, GFP; Yellow, merged. Unpaired *t*-test was used to analyze differences.

At different developmental stages of APP/PS1 transgenic mice, we examined the expression of Plek in mouse cortexes. Interestingly, Plek level was increased only at 12-month-old mice and had no significant differences at other stages (Figure 7A). Sdcbp2 had a little bit of increase in 6- to 12-month-old APP/PS1 mice when compared with that in age-matched WT mice, but without significant difference (Figure 7B). In PC12 cells, overexpression of Plek cannot increase the neurite length; however, when Plek was overexpressed together with miR-409-5p, it could partially rescue the miR-409-5p-induced neurite impairment (Figures 8A–D).

## Discussion

MiRNAs are abundantly expressed in brain and play essential roles in the maintenance of neuronal functions. During AD development, miRNA expression profile might indicate disease progress. In our study, we found that the level of miR-409-5p was stably downregulated from the early stage in APP/PS1 mice, which is consistent with the previous miRNA expression results of Aβ-treated primary hippocampal neurons (Schonrock et al., 2010). This suggested that miR-409-5p level may be regulated by Aβ and involved in AD pathogenesis. In our study, we found both in Aβ-treated cells and APP/PS mice, miR-409-5p was predominantly downregulated at a very early stage, indicating that it may potentially affect important biological pathways essential for proper brain function relevant to AD. How Aβ regulates miR-409-5p at early stages of AD is still unclear. The possible mechanisms include (1) the maturation process of miRNAs contains multiple steps, with multiple proteins affecting miRNA processing efficiency, which offer a plethora of regulatory options at both transcriptional and posttranscriptional levels (Winter et al., 2009). Aβ-related signal pathways may interfere with the multiple steps involved in the production of mature miRNAs. (2) miRNA turnover may be rapid, and Aβ may interfere with decay rate of miR-409-5p by inducing a rapid activation of miRNA-specific exoribonucleases, such as the 3′ → 5′ exonuclease SDN1 and the 5′ → 3′ exoribonuclease XRN-2 or other unknown nucleases (Ramachandran and Chen, 2008; Chatterjee and Grosshans, 2009; Schonrock et al., 2010).

Integrity of the cytoskeleton is a prerequisite for function and survival of neurons. The morphology of neuronal axons and dendrites is dependent on the dynamics of the cytoskeleton (Bettencourt da Cruz et al., 2005). Synapse loss and neuronal death are uneven within the AD (Zimmermann et al., 2005). Our data showed that miR-409-5p is highly neurotoxic. Overexpression of miR-409-5p inhibited the growth of neurites and reduced the cell viability of cultured neurons. As the KEGG pathway analyzed that the targets of miR-409-5p are enriched in the regulation of actin cytoskeleton and synapse, miR-409-5p may regulate neurite growth *via* its target genes, including Plek. Therefore, miR-409-5p may inhibit neurite outgrowth by targeting Plek and other cytoskeleton-related genes. How cytoskeleton regulation may be involved in AD progression needs more study.

The early downregulation of miR-409-5p seems like a self-protective reaction by neurons to alleviate the synaptic damage induced by Aβ. However, although Plek is also slightly increased from 6-month-old AD mice, it is not comparable with the miR-409-5p decrease. As Plek is an important protein in cytoskeleton reorganization, its expression change might be limited to some special subcellular locations such as dendrites and synapses. We still need more investigation to further understand the function of Plek in AD development.

Taken together, we found that the expression level of miR-409-5p was stably downregulated in the early stage of APP/PS1 mice. Aβ_1__–__42_ peptide significantly decreased the expression of miR-409-5p. The downregulation of miR-409-5p at early stages of AD may be a self-protective effect, and it may be used as an early biomarker of AD.

## Data Availability Statement

All datasets generated for this study are included in the article/Supplementary Material.

## Ethics Statement

The animal study was reviewed and approved by the Committee for the Ethics of Animal Experiments, Shenzhen Peking University – The Hong Kong University of Science and Technology Medical Center (SPHMC) (protocol number 2011-004).

## Author Contributions

JG carried out the molecular and cellular studies, participated in the animal experiments, and drafted the manuscript. YC carried out the immunostaining assays and revised the manuscript. NM, XY, YW, and BY participated in the animal experiments including tissue collection and RNA/protein extraction, and helped to revise the manuscript. JW conceived the study, participated in its design and coordination, and helped to draft the manuscript. All authors read and approved the final version of the manuscript.

## Conflict of Interest

The authors declare that the research was conducted in the absence of any commercial or financial relationships that could be construed as a potential conflict of interest.

## Footnotes

1 http://mirdb.org/miRDB/

2 http://www.microrna.org/microrna/home.do

3 http://snf-515788.vm.okeanos.grnet.gr/

4 http://www.targetscan.org/

5 http://bioinformatics.psb.ugent.be/webtools/Venn/

6 http://geneontology.org/

## Abbreviations

A β: beta amyloid peptide
AD: Alzheimer’s disease
MiR: MicroRNA
Plek: pleckstrin
Sdcbp2, syndecan binding Protein 2: syntenin2.
