# Supplementary material for: MiR-409-5p as a Regulator of Neurite Growth Is Down Regulated in APP/PS1 Murine Model of Alzheimer’s Disease
Source: Front Neurosci. 2019 Nov 28;13:1264. doi: 10.3389/fnins.2019.01264 (PMC6892840; doi:10.3389/fnins.2019.01264)
Supplement: Supplementary file 1 [file Data_Sheet_1.pdf]

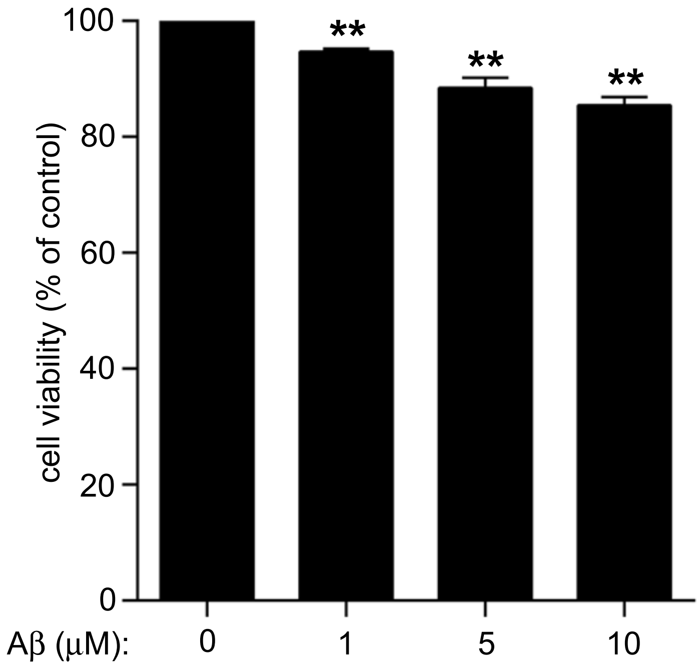

Supplementary figure 1. Dosage test of Aβ42 treatment in cell viability.

Primary-cultured hippocampal neurons were treated with different doses of Aβ42 for 24 hours. MTS/PMS assay was performed to evaluate the cell viability. The results were shown as the mean±SD (**$p < 0.01$, $n=3$).
