# Supplementary material for: MiR-409-5p as a Regulator of Neurite Growth Is Down Regulated in APP/PS1 Murine Model of Alzheimer’s Disease
Source: Front Neurosci. 2019 Nov 28;13:1264. doi: 10.3389/fnins.2019.01264 (PMC6892840; doi:10.3389/fnins.2019.01264)
Supplement: Supplementary file 2 [file Data_Sheet_2.pdf]

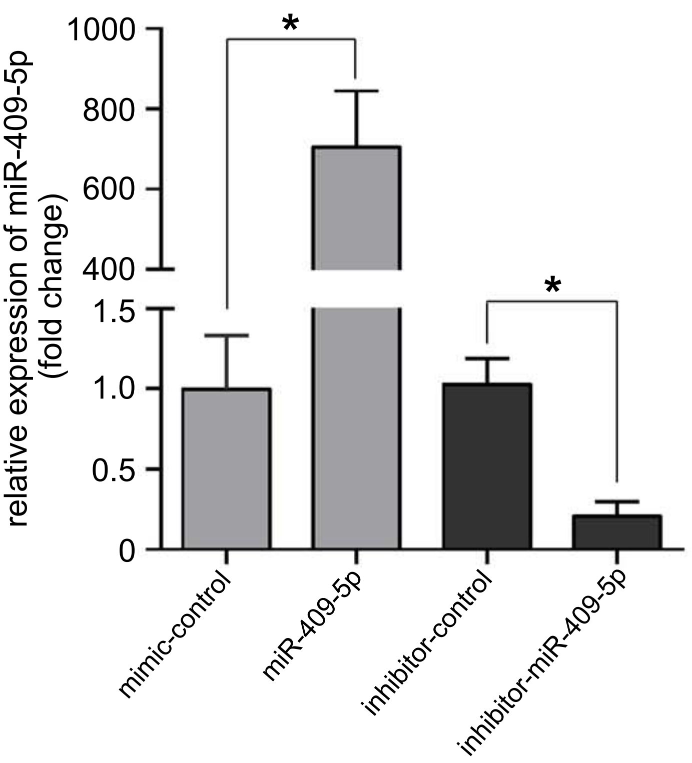

Supplementary figure 2. Transfection efficiency of miR-409-5p mimic and inhibitor.

PC12 cells were transfected with miR-409-5p mimic or inhibitor. Twenty-four hours later, relative expression level of miR-409-5p was examined by RT-qPCR. The results were shown as the mean±SD (*p< 0.05,n=3).
