# Supplementary material for: MiR-409-5p as a Regulator of Neurite Growth Is Down Regulated in APP/PS1 Murine Model of Alzheimer’s Disease
Source: Front Neurosci. 2019 Nov 28;13:1264. doi: 10.3389/fnins.2019.01264 (PMC6892840; doi:10.3389/fnins.2019.01264)
Supplement: Supplementary file 3 [file Data_Sheet_3.pdf]

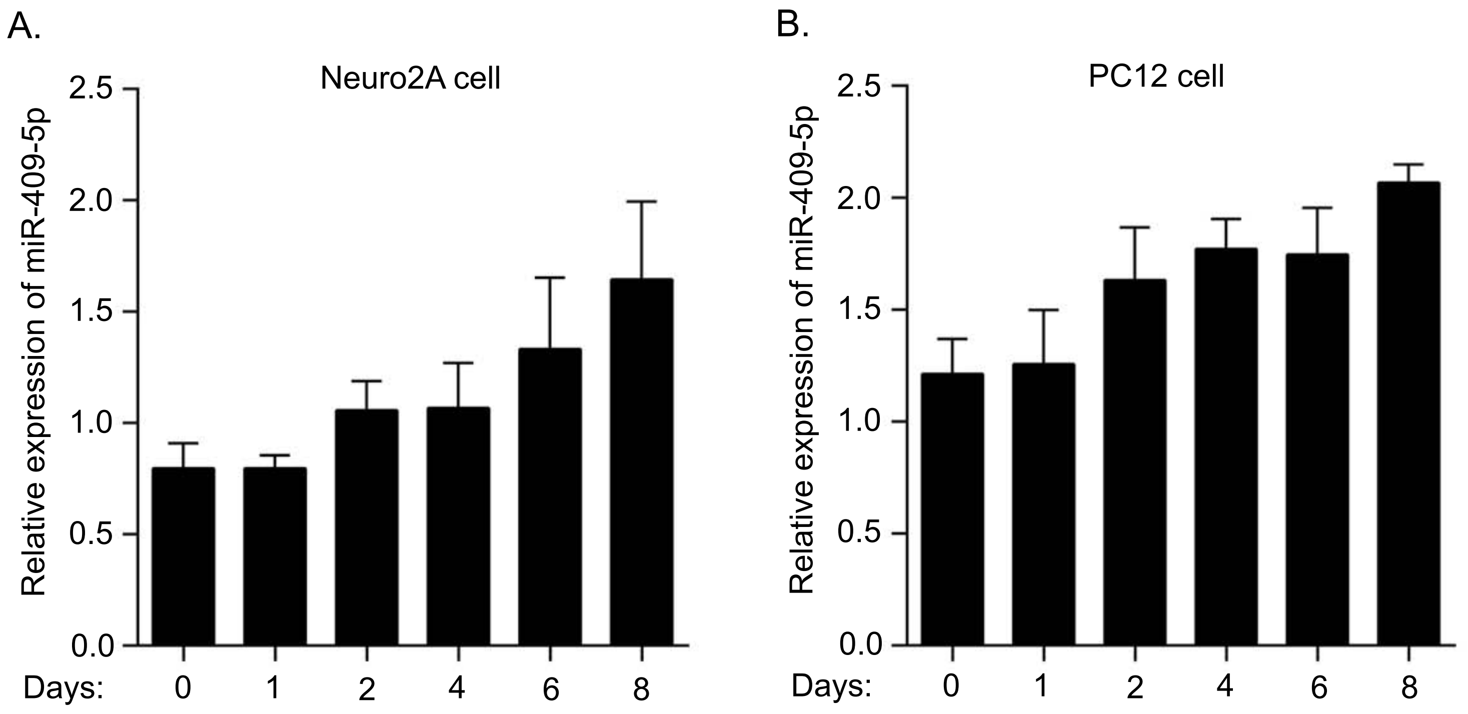


Supplementary figure 3. MiR-409-5p expression level along cell differentiation.

Neuro2A cells (A) or PC12 cells (B) were induced differentiation by RA or NGF, respectively. Relative expression level of miR-409-5p was examined by RT-qPCR at different time points.
