# Supplementary material for: MiR-409-5p as a Regulator of Neurite Growth Is Down Regulated in APP/PS1 Murine Model of Alzheimer’s Disease
Source: Front Neurosci. 2019 Nov 28;13:1264. doi: 10.3389/fnins.2019.01264 (PMC6892840; doi:10.3389/fnins.2019.01264)
Supplement: Supplementary file 4 [file Table_1.pdf]

Supplementary Table 1. Potential targets proteins of miR-409-5p by Venn tool.

Databases	Gene number	Gene Name
DIANA0.7+ miRDB+ miRanda+ targetscan	12	Kpna1
		Marcks
		Rimklb
		Stag2
		Lrp8
		Zdhhc9
		Gpm6a
		Tshz3
		Ankrd13c
		Gng12
		Cpsf6
		Zbtb34
DIANA0.7+ miRDB+ miRanda	8	Espnl
		Zic1
		Rnf19a
		Gsg2
		Crebzf
		Slfn4
		Kif1b
		Cbl
DIANA0.7+ miRanda+ targetscan	7	Ube2l3
		Cbln2
		Rab5c
		Znf512b
		Acvr2a
		Nr1i3
		Aplp2
DIANA0.7+ miRDB +targetscan	2	Trps1
		Suv420h1
miRDB+ miRanda+ targetscan	11	Ubxn4
		Gcdh
		Adam10
		Rasl12
		Pi4kb
		Mkrn1
		Serf2
		Ppara
		Usp7
		Kcna1
		Arid4b
DIANA0.7+miRanda	26	Utrn

		Camk4
		Rnf24
		Zfp526
		Synj1
		Lemd3
		Trim15
		Kdm5c
		Fbn2
		Sltm
		Ell2
		Naf1
		Fam19a2
		Nbea
		Uhmk1
		Gm8773
		Otud4
		Tmem108
		Arpc4
		Gpm6b
		Lrrtm3
		Psd3
		A330008L17Rik
		Tmem30a
		Nrf1
		Grm3
DIANA0.7+miRDB	1	Srsf2
DIANA0.7+targetscan	4	Luzp1
		Plagl1
		Zfp652
		Khdrbs1
miRDB miRanda	38	
		Dram2
		Macf1
		Mrpl41
		Nudcd3
		Fam64a
		Tada1
		Rab23
		Anxa7
		Mtmr3
		Camta1
		Vdac2
		Shpk
		Samd4b

		Rsbn1
		Tfam
		Ppp4r4
		Gls
		Zfp382
		Tmc5
		Mc2r
		Ugcg
		Trpm3
		Plek
		Gm94
		Slc26a1
		Mlh3
		Ptk7
		Kcnh8
		Nolc1
		Rbl1
		Kctd10
		Zic3
		Uba6
		Sdcbp2
		Nudt18
		Ifrd1
		Grsf1
miRanda targetscan	28	Kcnd3
		Kif21b
		Akap1
		Slc1a1
		Fus
		Tmigd1
		Arap3
		Hmbs
		Hapln4
		Ar
		Zfp384
		Itgb4
		Akirin2
		Tanc2
		Fyn
		Snx27
		Pdap1
		Pgf
		Uck2
		Astn2

		Arhgap26<br>Tlx1<br>Ptpn7<br>Vrk1<br>Rnd3<br>Ctbp1<br>Elk1<br>Nrl
miRDB targetscan	2	Nadk2<br>Mtss1
